# Editorial: In the footsteps of the prosomeric model

**DOI:** 10.3389/fnana.2022.1010058

**Published:** 2022-08-23

**Authors:** José L. Ferran, Matías Hidalgo-Sánchez, Eduardo Puelles

**Affiliations:** ^1^Department of Human Anatomy and Psychobiology, School of Medicine, University of Murcia, Murcia, Spain; ^2^Institute of Biomedical Research of Murcia—IMIB, Virgen de la Arrixaca University Hospital, El Palmar, Spain; ^3^Departamento de Biología Celular, Facultad de Ciencias, Universidad de Extremadura, Badajoz, Spain; ^4^Instituto de Neurociencias, Consejo Superior de Investigaciones Científicas, Universidad Miguel Hernández de Elche, Elche, Spain

**Keywords:** neuromeres, secondary prosencephalon, mesomeres, rhombomeres, neuromeric model

Two paradigms on fundamental brain structure have disputed primacy during the last 100 years: the neuromeric model, which attends to transversally bulging neural tube subdivisions or neuromeres (function as intermediate units of developmental molecular identities), and the columnar model, which attends to adult longitudinal ventricular sulci, held to separate basic sensorimotor/viscerosomatic functional columns (also in the forebrain). The columnar model was predominant up to the birth of molecular neurobiology. However, molecular and causal exploration of the developing brain, and progress in correlative evolutionary studies, showed that the columnar model has poor developmental explanatory value and, worse, is inconsistent with accruing molecular and experimental data (e.g., definition of axial and other spatial directions inconsistent with the causal molecular background; difficulty for ascribing meaning to observed gene expression patterns; adult sulci found not developmentally relevant, being unrelated to gene expression patterns). Contrarily, success in assimilating such novel data put the neuromeric model back into orbit, singularly in the updated format of the prosomeric model of Puelles and Rubenstein (1993, 2003, 2015) and Puelles (2017, 2018). This model defines the axial dimension of the whole neural tube (which eventually subdivides into a constant set of neuromeres, after manifesting first forebrain, hindbrain, and spinal tagmata, and their proneuromeric subdivisions) as related to both rostrocaudal prechordal plate signals (strongest at the acroterminal rostral forebrain midline) and ventrodorsal (ventralizing) notochordal signals (strongest at the floor plate; antagonistic with dorsalizing roof plate signals). Various secondary morphogenetic axial bending phenomena and some areal vesicular outgrowths are considered topologically invariant as regards relevant molecular pattern and need to be kept in mind. Intersecting longitudinal and transverse neural tube zones proceed interactively to define specific histogenetic units or progenitor domains with unique molecular profiles, which collectively build the complex brain. The basic pattern is held to be common to all vertebrates, irrespective of significant morphogenetic variations and eventual further regionalization in some lineages. Early molecular regionalization leads to patterned proliferation, position-dependent neurogenesis of different cell types with characteristic migratory and adhesive properties, guided axonal navigation, and organized synaptogenesis. Functions finally start to emerge as the neural system matures.

The leading figure in the definition and updating of the prosomeric model, now predominant in evo-devo studies, is Puelles. He has been significantly supported by J. L. R. Rubenstein among other colleagues since the nineties (a still continuing collaboration). He is still active as Professor Emeritus at the University of Murcia, Spain. We want to honor him by reviewing in this special issue from different perspectives how the prosomeric model has insightfully aided us in understanding the different regions of the brain of vertebrates in connection with the complex morphologic meaning of gene patterns. His detailed models of the forebrain (midbrain, diencephalon, hypothalamus, subpallium, pallium, amygdala) and the hindbrain (isthmus, cerebellum, prepontine, pontine, retropontine and medulla oblongata regions) are first-rate instruments to explore patterning, histogenesis, morphogenesis and evolution of the brain, including connectivity, synaptogenesis and malformations.

This special issue includes Puelles's own account explaining how he got involved in the prosomeric model from the beginning. He provides a personal and entertaining story in which he tells from his own perspective how he got involved in developing the prosomeric model at each stage of his scientific career. Beginning with his early formative years in Seville, passing through his experience in Cadix, to finally settle in Murcia, highlighting his interaction with J.L.R. Rubenstein as a key step in the birth of the prosomeric model. The self-taught scientific stance of Puelles stands out from the manuscript, as well as the large number of hours dedicated to wide reading in a field that was “thought to be solved,” an experience that allowed him to deepen his conceptual vision.

Some studies in this issue explored early antero-posterior neural tube patterning, axon guidance, or the distribution of specific gene expression patterns throughout the brain using prosomeric landmarks. During the formation of the neuromeric boundaries along the rostrocaudal axis of the neural tube, the action of signaling centers is required as a source of diffusible morphogens that act in neighboring territories. The midbrain-hindbrain (MH) constriction is a well-studied model involved in the specification of both midbrain and isthmo-cerebellar primordia. Hidalgo-Sánchez et al. present an exhaustive review of the cellular and molecular mechanisms that specify the MH territory *via* the establishment of the secondary organizer. Callejas-Marin et al. used a *Gli2* knockout mouse to determine the role of *Shh* in the development of thalamocortical projections. They found that if Gli2-mediated *Shh* signaling is impaired, the medial ganglionic eminence and the lateral ganglionic eminence are not correctly specified. Such altered specification may explain the difficulty of thalamo-cortical axons to cross the subpallium to reach the cortex. The prosomeric model also became a highly resolutive tool to define the precise position of gene/protein expression patterns of neurons distributed in large or small brain regions. The study by Pombal et al. describes in remarkable detail the distribution of NADPH-d (nicotinamide adenine dinucleotide phosphate-diaphorase) in the adult lamprey brain following neuromeric landmarks. These results are compared with the distribution found in gnathostomes. According to this study, the NADPH-d components observed in the adult lamprey are significantly different from those described in larval stages, with some positive components topologically comparable to those observed in other vertebrates.

Some manuscripts in this issue were devoted to the study of telencephalic derivatives, another important domain illuminated by the collaborative work of Puelles and Rubenstein. The genoarchitecture of the feline telencephalon was analyzed by Siskos et al. in this issue. Using the prosomeric model, the authors molecularly define the pallial and subpallial domains and demonstrate that the mapping from E26/E27 cats is comparable to that of E13.5/E14 mice. The authors also confirmed in cats the tetrapartite model of Puelles et al. (2000, 2017) as well as the main subpallial compartments (Puelles et al., 2013, 2016). The authors thus provide a gyrencephalic brain model to study key telencephalic phylogenetic questions. Fang et al. analyzed the birthdate pattern of *Nurr1*-positive claustral neurons in the rat and the progress of claustral neurogenesis throughout development. *Nurr1*-positive neurons located in the claustrum are part of the lateral pallium domain of the prosomeric model. The authors combined EdU injections with *in situ* hybridization for *Nurr1* throughout the developmental period of the rat brain. They identified the birth dates for the neurons of the dorsal endopiriform, ventral claustrum, and dorsal claustrum nuclei, and the cortical deep and superficial layers. Aerts and Seuntjens wrote a detailed review of the developmental origin of the nuclei that are part of the amygdala complex. The authors provide a summary of previous reports, including some reinterpretations derived from the recent pallial concentric ring theory and the radial histogenetic model of the pallial amygdala (Puelles et al., 2019; Garcia-Calero et al., 2020). Furthermore, a rational analysis of migratory routes related to transcription factors and guidance signals is provided in this study. Metwalli et al. identify the telencephalo-opto-hypothalamic (TOH) domain in sauropsids. The TOH was recently identified in mice as a new radial embryonic division postulated to be the source of the majority of glutamatergic neurons in the medial extended amygdala. The study published in this issue examines the coexpression of *Foxg1* and *Otp* transcription factors to identify the same domain in chicken and lizard. This work highlights the complexity of the hypothalamic-telencephalic transition, opening new challenging questions to be explored in further studies.

Other studies in this special issue explored different questions related to the hypothalamic or the pretectal region using the prosomeric model for anatomical, comparative and/or causal analysis of these brain regions. Santos-Durán et al. performed a detailed analysis of the hypothalamic region of the catshark Scyliorhinus canicula (Chondrichthyans) at developmental stage 32. The authors used the prosomeric model to interpret the adult identity and location of the main hypothalamic nuclei and postulate some mammalian homologies. This work provides a framework for phylogenetic and ontogenetic hypothalamus studies in cartilaginous fishes. Conventional pharmacological, electrophysiological and neurochemical studies of the hypothalamic region usually visualize this region in rats under columnar model landmarks (implying an orthogonal axis formulation). In this issue, Bilbao et al. performed an immunohistochemical mapping of hypothalamic neurons expressing tyrosine hydroxylase based on the modern prosomeric model. This neuromeric mapping elucidates the relationships of TH-positive hypothalamic neurons, defining the precise location of the TH-positive cells in relation to potential sources of morphogenetic signals. This analysis demonstrates that the prosomeric model is a useful tool to postulate causal mechanisms that specify hypothalamic cell types, in contrast with the silence of the columnar model in this regard (e.g., Swanson, 2012). Brozko et al. identify derivatives of the three rostrocaudal pretectal progenitor domains postulated previously in neuromeric studies (Ferran et al., 2007), examining zebrafish early developmental stages. This study develops a prosomeric molecular map of the zebrafish diencephalic region identifying the main pretectal partitions at 48 hpf. Using this anatomical description based on the prosomeric model they revealed an instructive role of Wnt signals controlling the size of GABAergic and glutamatergic clusters during pretectal development in zebrafish.

Finally, some works in this topic prove to be highly resolutive in understanding the hindbrain region using the prosomeric model. According to the updated neuromeric model, the hindbrain is subdivided into twelve anteroposterior segmental units called rhombomeres (r0–r11; Puelles, 2018). Classical anatomic studies recognized oral, interpolar, and caudal subnuclei in the spinal column of the trigeminal nucleus. García-Guillén et al. examined the molecular segmentation of the spinal trigeminal nucleus consistently with the prosomeric model. This study identifies the spinal trigeminal nucleus as a plurisegmental structure extending from r4 to r11 in mice. Further, the authors recognize unique molecular signatures for each segmental neuromeric partition of the spinal trigeminal nucleus, as predicted by the model. The hindbrain region was also analyzed under the prosomeric viewpoint in the systematic review by Hirsch et al. The authors study the subtype class A (Olig3-expressing) and B (Lbx1-expressing) dorsal interneurons positioned in specific dorsoventral subdomains of the alar plate along the longitudinal dimension of the hindbrain. Transgenic and mutant animal models revealed that, despite the similar genetic profile of dorsal interneurons along the antero-posterior dimension of the hindbrain, they contribute to specific nuclei according to their rhombomeric origin. The vestibular sensory column of the hindbrain was reviewed by Diaz and Glover from a neuromeric perspective. The authors performed an exhaustive review of previous studies that analyzed the morphological and functional organization of the vestibular complex from the prosomeric point of view. The study highlights that the antero-posterior subdivision pattern results in rhombomeres with unique molecular signatures, which can be correlated with hodologically defined groups of vestibular neurons that are either monosegmental or plurisegmental in origin.

The studies in this special issue are representative examples demonstrating that the prosomeric model is a powerful tool for exploring key questions about the brain that require precise anatomical characterizations. Modern neurobiology will be enriched from prosomeric analysis by providing the appropriate frameworks to explore the causal mechanisms that underpin brain development. In addition, genoarchitectonic studies (Puelles and Ferran, 2012) based on the prosomeric model are benefiting evolutionary studies that require cutting-edge comparative anatomy to locate homologous nuclei, regions or layers between vertebrates. The prosomeric model, to which Puelles has been one of its main supporters, is a key instrument for experimental development that, ultimately, contributes to understanding the functioning of the brain by providing high-definition anatomical landmarks.

## Author contributions

JF, MH-S, and EP: writing, review, and editing. All authors contributed to the article and approved the submitted version.

## Funding

This study was funded by the Spanish Ministry of Science, Innovation, and Universities (MCIU), State Research Agency (AEI) and European Regional Development Fund (FEDER); PGC2018-098229-B-100 and by Séneca Foundation (19904/GERM/15) to JF; by Junta de Extremadura, Grant/Award No. GR21167 (MH-S); Junta de Extremadura, Fondo Europeo de Desarrollo Regional, Una manera de hacer Europa, Grant/Award No. IB18046 to MH-S and by the MINECO/AEI/FEDER (BFU2013-48230) to EP, and the Institute of Neurosciences is a Center of Excellence Severo Ochoa (SEV-2017-0723).

## Conflict of interest

The authors declare that the research was conducted in the absence of any commercial or financial relationships that could be construed as a potential conflict of interest.

## Publisher's note

All claims expressed in this article are solely those of the authors and do not necessarily represent those of their affiliated organizations, or those of the publisher, the editors and the reviewers. Any product that may be evaluated in this article, or claim that may be made by its manufacturer, is not guaranteed or endorsed by the publisher.
